# Aberrant CD200/CD200R1 expression contributes to painful synovium hyperplasia in a patient with primary hypertrophic osteoarthropathy

**DOI:** 10.1007/s00296-013-2732-1

**Published:** 2013-04-18

**Authors:** Yan Ren, Xiao-mei Leng, Bo Yang, Xuan Zhang

**Affiliations:** 1 Department of Rheumatology, Peking Union Medical College Hospital, Peking Union Medical College and Chinese Academy of Medical Science, Beijing, 100730 China; 2 Department of Orthopaedics, Peking Union Medical College Hospital, Peking Union Medical College and Chinese Academy of Medical Science, Beijing, 100730 China; 3 Department of Health Care, China-Japan Friendship Hospital 2 Yinghua Dongjie, Hepingli, Beijing, 100029 China

**Keywords:** Primary hypertrophic osteoarthropathy, CD200/CD200R1, Endothelial activity

## Abstract

We present a case of hypertrophic osteoarthropathy (PHO), with painful synovium hyperplasia involving both knees that was refractory to corticosteroid treatment. His rheumatoid factor and anti-CCP antibody was negative, and his serum ESR and CRP level was within normal range. Histological examination of the synovium obtained from his right knee revealed endothelial hyperplasia and vascular thickening without inflammation that was in association with aberrant expression of CD200/CD200R1, which highlighted the importance of aberrant CD200/CD200R1 in the regulation of the endothelial activation that contributed to the development of synovium hyperplasia in this PHO patient.

## Introduction

Hypertrophic osteoarthropathy (PHO) is a rare disease of unknown aetiology characterized by hypertrophic skin changes, clubbing fingers and periostitis [1]. Synovial effusion is less common usually symptomless. If painful, it usually responds well to non-steroidal anti-inflammatory drugs (NSAIDs). Here, we present a case of a PHO patient with severe pain and swelling of both knees which were refractory to NSAIDs and corticosteroid treatment. Histological examination of the synovium obtained from his knee demonstrated endothelial hyperplasia and vascular thickening without inflammation together with aberrant CD200/CD200R1 expression.

## Case report

**Fig. 1:**
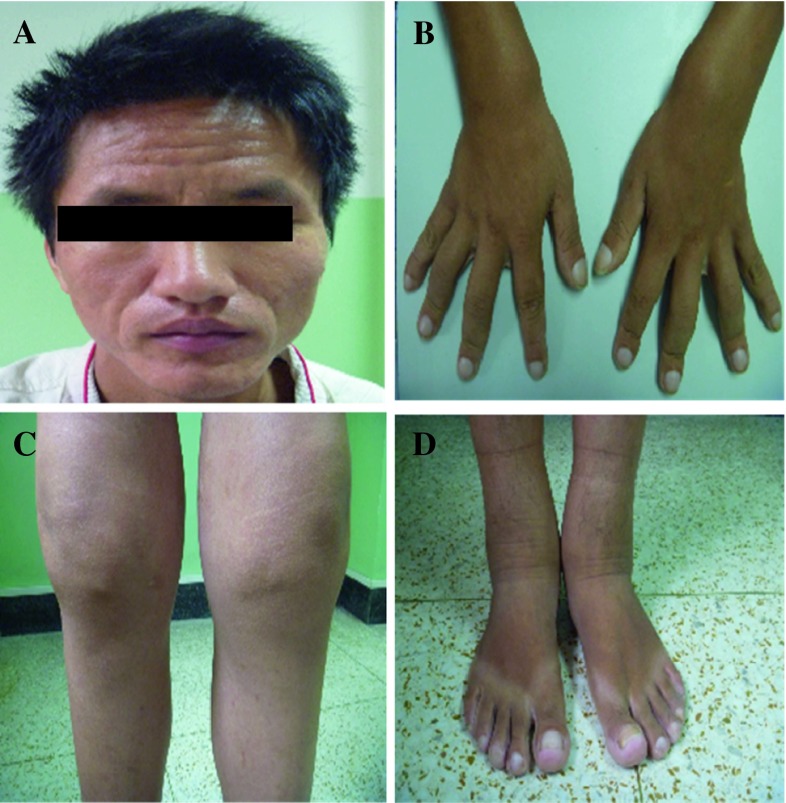
Clinical findings of the PHO patient with hyperplastic synovium. **a** Furrowy forehead; **b** clubbing fingers; **c** bilateral knee effusion; **d** ankles with oedema

**Fig. 2:**
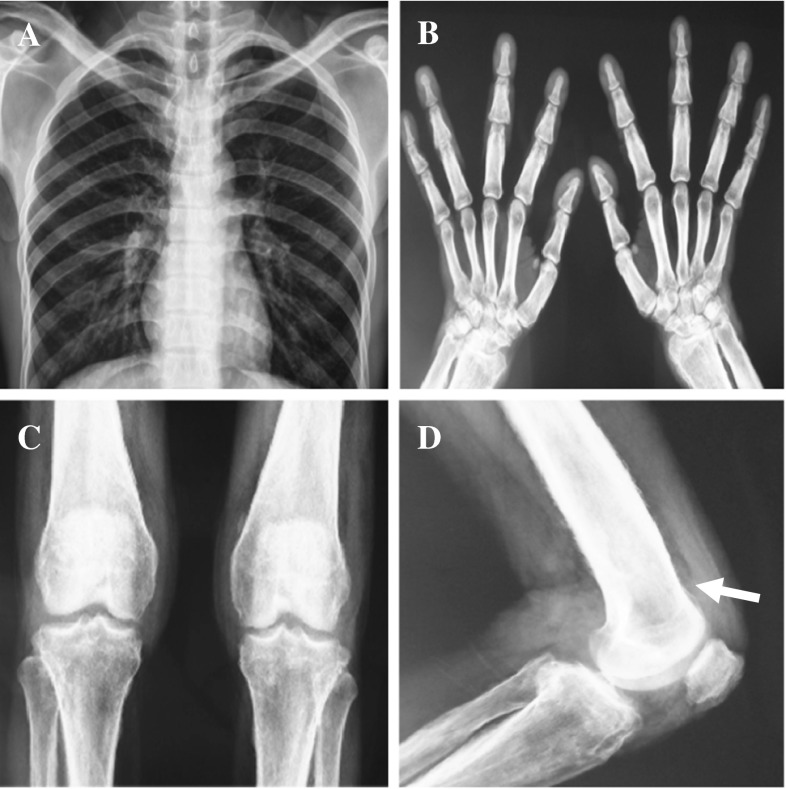
Radiography of the PHO patient with hyperplastic synovium. **a** Normal X-ray; **b** both hands showed periostosis; **c** both legs showed periostitis in the proximal tibia; **d** pronounced periosteal reaction at the distal end of femur (*arrow*)

**Fig. 3:**
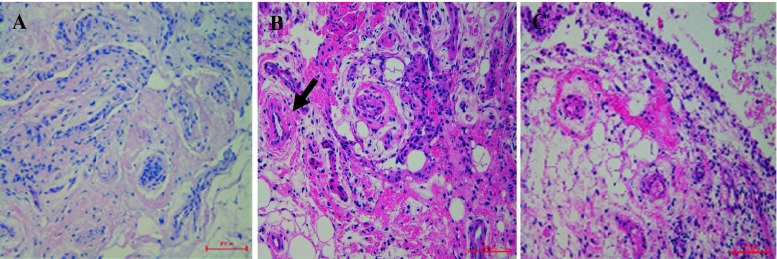
Synovium histology. This PHO patient with hyperplastic synovium had endothelial hyperplasia and vascular thickening (*arrow*) without inflammation (Haematoxylin and eosin staining, original magnification × 200). The histology of the synovium from a healthy control (HC) and a PHO patient without hypertrophic synovium was also included as controls

**Fig. 4:**
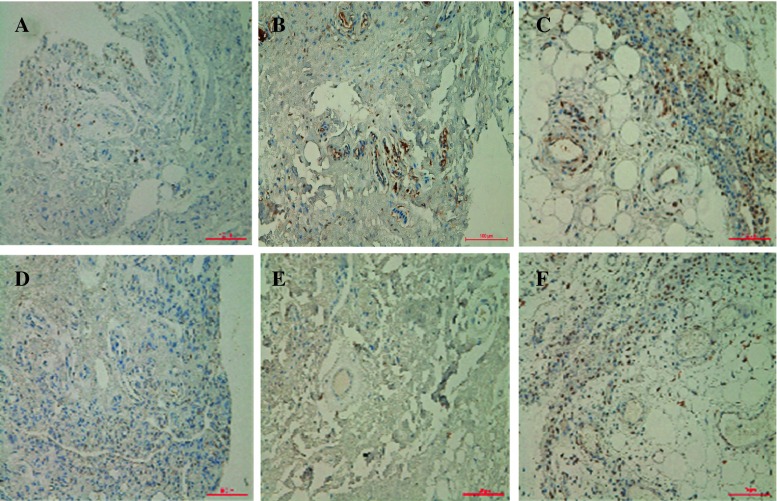
Immunohistochemistry examination of the PHO patient with hyperplastic synovium demonstrated less expression of CD200 (**b**) and CD200R1 (**e**) in the synovium of the PHO with hypertrophic synovium compared with that of healthy control (HC, **a**, **d**) and a PHO patient without synovium hyperplasia (**c**, **f**, original magnification × 200)

This is a 28-year-old Chinese man with intermittent arthralgia affecting multiple joints after strenuous work for 10 years. He complained of increasing disabling pain and swelling of both knees in recent 6 months. He also had profuse sweating of his palms and soles. He started NSAIDs 5 years ago with little symptom effect. His symptoms did not respond to 30 mg/day of prednisone either. In recent 3 months, he had a weight loss of around 5 kg and developed hyperhidrosis. Physical examination revealed pachydermia with a thickening of his forehead folds. He had palmoplantar hyperhidrosis and cutis verticis gyrata, severe clubbing of all his fingers and symmetrical effusion in his knees and ankles (Fig. 1). Examination of his chest and abdomen was unremarkable. His laboratory tests were as follows: ESR 13 mm/h, CRP < 3.0 mg/l, ANA(−), anti-ENA(−), RF(−) and anti-CCP(−). His liver, kidneys and growth hormone tests were within normal ranges. The X-ray of his bones revealed irregular periosteal hypertrophy with bone formation affecting his long bones and phalanges bilaterally (Fig. 2). There was no abnormal finding on his chest radiograph, abdominal ultrasound, echocardiograph and gastroendoscopy examinations. The synovial fluid obtained from his knee was non-inflammatory. On gross examination, the synovial biopsy sample from his right knee showed venous dilatation, and histological examination demonstrated endothelial hyperplasia and vascular thickening without any inflammation (Fig. 3). Immunohistochemical staining revealed that the proportion of CD200+ and CD200R1+ cells were down-regulated compared with that from healthy control and a PHO patient without synovium hyperplasia. In addition, not like the CD200+ cells in the PHO without synovium hyperplasia, which was mainly identified in the lining layer and to a lesser extent in the sublining layer, the CD200+ cells in the PHO patient with hyperplastic synovium extended to the vascular endothelial regions, whereas CD200R1+ cells were hardly detected (Fig. 4).

## Discussion

PHO, or touraine-solente-gole syndrome (TSG), which is often familial and affects predominantly male, was firstly reported by Friedrich in 1868 [2]. It can be divided into primary and secondary subtypes. The case presented here is a complete form of pachydermoperiostosis (PDP), the primary form of PHO, since he had hyperostosis, finger clubbing and pachydermia without any underlying cardiovascular, pulmonary, liver, intestinal or mediastinal diseases. Importantly, PDP with refractory and painful synovium hyperplasia, as demonstrated in our patient, is rarely reported. As the synovial fluid in this case is non-inflammatory, we need to explore why he had painful synovial hyperplasia. Thereby, we performed synovial biopsy on his right knee and found venous dilatation. The histological examination demonstrated endothelial hyperplasia and vascular thickening in his synovium compared with that from healthy and PHO control, highlighting the pathology of vascular neoformation and microangiopathy in the hyperplastic synovium of this PHO case.

CD200 is a type I transmembrane glycoprotein belonging to the immunoglobulin superfamily, is widely distributed in various tissues including the vascular endothelium [3], is known for its immunoregulatory function in immune response [4–6] and may participate in the regulation of the endothelial function [7]. In our previous study, we demonstrated that there was an abnormal expression of CD200 and CD200R1 in RA patients and this abnormality contributes to the immunopathogenesis of RA. However, our knowledge of the role of the CD200/CD200R1 axis in human PHO is limited. Given that apparent painful synovial hyperplasia existed either in RA or this PHO case, it would be very interesting to explore whether this pathway may exert immunosuppressive functions on non-inflammatory disease, especially in PHO. We therefore assessed the distribution and extent of CD200 in the synovium of this PHO case, and we found that there were less CD200+ and CD200R1+ cells in his synovium compared to HC and the PHO patient without synovium hyperplasia. Of note, not like the CD200+ cells in the PHO patient without synovium hyperplasia, which was mainly identified in the lining layer and to a lesser extent in the sublining layer, the CD200+ cells in this PHO patient with hyperplastic synovium extended to the vascular endothelial regions, whereas CD200R1+ cells were hardly detected. The explanation for this discrepancy is not certain, but the refractory synovial effusion observed in this PHO was associated with aberrant local expression of CD200/CD200R1 that might led to abnormal vascular neoformation and microangiopathy since the following ultrastructural examination verified increased activation of endothelial cells at the sites of capillaries and arteriovenous anastomoses of this PHO case, highlighting the importance of CD200-CD200R1 interaction in the regulation of the endothelial activation, which might result in increased susceptibility to perivascular lymphocytic infiltrate, and ultimately lead to fluid effusion. Furthermore, availabilities of endothelial CD200 and CD200R1 in the blood vessels may contribute to the unequal predisposition of pathological conditions such as enhanced vascular dysfunction and alterative permeability.

In summary, we reported a case of PHO with synovium hyperplasia which was refractory to treatment and highlighted the importance of aberrant CD200/CD200R1 in the regulation of the endothelial activation that contribute to the development of synovium hyperplasia.
